# Aspectual Fertility Variation and Its Effect on Gene Diversity of Seeds in Natural Stands of Taurus Cedar (*Cedrus libani* A. Rich.)

**DOI:** 10.1155/2017/2960624

**Published:** 2017-01-09

**Authors:** Nilüfer Yazici, Nebi Bilir

**Affiliations:** Forestry Faculty, Suleyman Demirel University, Isparta, TR-32260, Turkey

## Abstract

There are many environmental and biological factors on forestry practices as known. Aspect called as slope faces is one of the most important environmental factors in these practices because of its easy application for managers. Fertility variation defined as an individual ability to give progeny and gene diversity estimated based on effective number of parents were investigated as the proportion of numbers of cones counted from individuals in natural stands sampled aspectual of Taurus cedar (*Cedrus libani* A. Rich.) for three consecutive years. The averages of cone number were 19.4, 47.2, and 75.5 for the years. It was the highest in flat (23.5) for 2013, in south (92.1) for 2014, and in flat (95.7) for 2015, while it was lowest in south (16.3), in east (18.2), and in north (39.4) for the years, respectively. Significant correlations (*p* ≤ 0.01) were estimated among years for cone production in polled aspect. Estimated fertility variations changed for the years and stands. It could be generally acceptable level for typical natural stands except of west of 2014. Fertility variations were 1.55, 3.05, and 1.64 in polled stands for the years. Gene diversity was 0.99 for the years in polled stands. North aspect could be taken into consideration in establishment and selection of seed sources and gene conservation areas based on fertility variation and gene diversity.

## 1. Introduction

There are many environmental (i.e., soil properties, climate, altitude, aspect, and slope) and biological (i.e., genetic structure of population or individual plant) factors on forestry practices. Aspect defining the compass direction that a slope faces is one of the most important environmental factors because of its easy application and cheap determination for these practices such as silvicultural, watershed, and other forestry operations for managers because of its effect on soil and forest formation and habitat. Aspect can have a strong influence on temperature. This is because of the angle of the sun in the northern and southern hemispheres which is less than 90 degrees or directly overhead. For instance, aspect is a criterion in selection and establishment of seed sources and selection of gene conservation areas [1] and their managements. However, aspectual fertility variation and gene diversity have not been investigated or reported in the species or other plant species until now, while the environmental factors were related to fertility variation and gene diversity based on reproductive output (e.g., [2–5]). Fertility is defined as an individual ability to give progeny (i.e., reproductive success). Estimation of fertility variation among genotypes is one of the important tools used in different purposes such as gene conservation, seed production programs, managing forest genetic resources, and evolutional and genetic management of populations for plant breeding [2, 6–8] based on sustainable forestry. It was known that there were many environmental and genetical factors on reproductive characters and variation among population and within population. However, while many studies (most of them on estimation practices) have been carried out on fertility variation (e.g., [4, 9–14]), environmental studies on fertility variation and its related parameters (i.e., effective number of parents and gene diversity) are very limited [2, 3].

The main objective of the present study is to evaluate the fertility variation in selected aspectual natural stands of Taurus cedar basis of cone production over a period of three consecutive years and to estimate the effective number of parents and gene diversity in the stands. The impacts of aspect on fertility variation and gene diversity were also discussed for future forestry practices of the species.

## 2. Materials and Methods

Five different natural stands called also aspectual populations located at latitude between 37°49′ and 37°53′ N, longitude between 31°15′ and 31°20′ E, and average elevation of 1600 m of Taurus cedar were sampled aspectually (flat, north, south, west, and east aspects) in Isparta region at southern part of Turkey. Numbers of mature cone were counted from the same numbered thirty trees selected phenotypically during three consecutive years (2013–2015).

Cone fertility variation (Ψ_*C*_) was estimated based on individual cone production as [3]

(1) $$\begin{array}{c} \Psi_{C}=N\overset{N}{\underset{i=1}{\sum }}{Con}_{i}^{2}={CV}_{C}^{2}+1, \end{array}$$

where *N* is the census number; Con_*i*_ is the cone fertility of the* i*th individual. CV_*C*_ is the coefficient of variation in total fertility. In this paper, the fertility of* i*th individual was estimated by the proportion of cone production in the population. Therefore, the cone fertility represents the total contribution as zygotic parents.

The effective number of parents (*N*_*p*_) was estimated as [8]

(2) $$\begin{array}{c} N_{p}=\frac{N}{\Psi_{C}}. \end{array}$$

The relative effective number of parents (*N*_*r*_) was estimated relatively compared to the census number as *N*_*r*_ = *N*_*p*_/*N*.

Gene diversity (GD) was estimated based on effective number of parents (*N*_*p*_) as [9]

(3) $$\begin{array}{c} GD=1-\frac{0.5}{N_{P}}. \end{array}$$

One-way ANOVA was performed in order to elucidate the differences between years as well as among stands:

(4) $$\begin{array}{c} Y_{ij}=\mu +P_{i}+e_{ij}, \end{array}$$

where *Y*_*ij*_ is the observation from the* j*th tree of the* i*th population/population, *μ* is overall mean, *P*_*i*_ is the random effect of the* i*th population, and *e*_*ij*_ is random error.

Phenotypic correlations (*r*_*p*_) [15] among years for cone production were calculated using SPSS statistical package program as

(5) $$\begin{array}{c} r_{p}=\frac{{COV}_{f\left(x,y\right)}}{\sqrt{{\sigma^{2}}_{f\left(x\right)}}\sqrt{{\sigma^{2}}_{f\left(y\right)}}}. \end{array}$$

COV_*f*(*x*, *y*)_ is the covariance between the characters *x* and *y*; *σ*^2^_*f*(*x*)_ and *σ*^2^_*f*(*y*)_ are the phenotypic variances of character *x* and character *y*, respectively.

## 3. Results and Discussion

### 3.1. Cone Production

**Figure 1:**
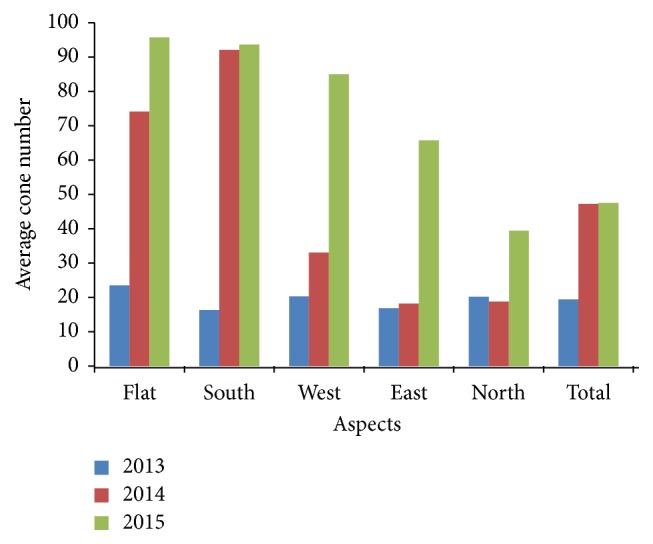
Averages of cone production for aspectual populations and years.

There were large differences among aspects and years and also within aspect and year for cone production (Table 1 and Figure 1).

The averages of cone number were 19.4, 47.2, and 75.5 for the years. Third year was good seed year by average of 75.5 cones per tree of the species (Table 1). The results' well accordance with that good seed year of the species in natural stands was once in three years [16]. The average of cone number was the highest in flat (23.5) for 2013, in south (92.1) for 2014, and in flat (95.7) for 2015, while it was lowest in south (16.3), in east (18.2), and in north (39.4) for the years, respectively (Table 1). The most abundant six trees (20% of total tree) produced 36.1%, 44.2%, 50.4%, 49.1%, and 38.3% of total cone production in the flat, south, west, east, and north populations for 2013, respectively, while it was 60.5%, 56%, 69.2%, 58%, and 41.4% for 2014 and 39.2%, 50.1%, 35.3%, 50.5%, and 52.6% for 2015. Besides, 23 individual trees did not produce any cone during observation in combined population and years; it was the highest in east population by 11 trees in polled years. The differences were also higher within aspect and year (Figure 1). For instance, coefficient of variation of cone production was the highest (105.0%  and 175.2%) in west for 2013 and 2014. It was the lowest (52.7%) in the same aspect for 2015. It was calculated as 79.4%, 155.3%, and 81.0% for the years in polled populations. South aspect produced the highest cone (67.4) by the highest coefficient of variation of cone production (93.5%) for polled years. They were the lowest in north aspect (26.1% and 63.6%) (Table 1). These variations showed importance of stand/aspect and individual selection for higher cone production. Statistically significant differences (*p* ≤ 0.05) were found among stands for cone productions of 2014 and 2015 based on results of analysis of variance, while it was opposite for 2013. Large differences in fertility were reported among trees in natural populations of different forest tree species (e.g., [17, 18]) and among clones in seed orchards [8, 17, 19, 20]. Differences in gamete contribution could be genetic [21] and could be influenced by environmental factors [22] such as water regime of aspects [23] and its effect on soil characters. Environmental factors were more important than genetic constitution for the reproductive variation in* Pinus sylvestris* (e.g., [4, 5]). It was also found that age, elevation, and crown closure were important factors in seed yield of* Pinus brutia *[24]. Altitude, age, and environmental variation, mainly in soil properties, could be effective on the observed variation in fruiting and seed set within each population in the natural forest [17].

There were positive and significant (*p* ≤ 0.01) correlations among years for cone production in the stands (Table 2). The results had well accordance with other forest tree species carried out on* Pinus brutia* [2].

### 3.2. Fertility Variation, Effective Number of Parents, and Gene Diversity

**Figure 2:**
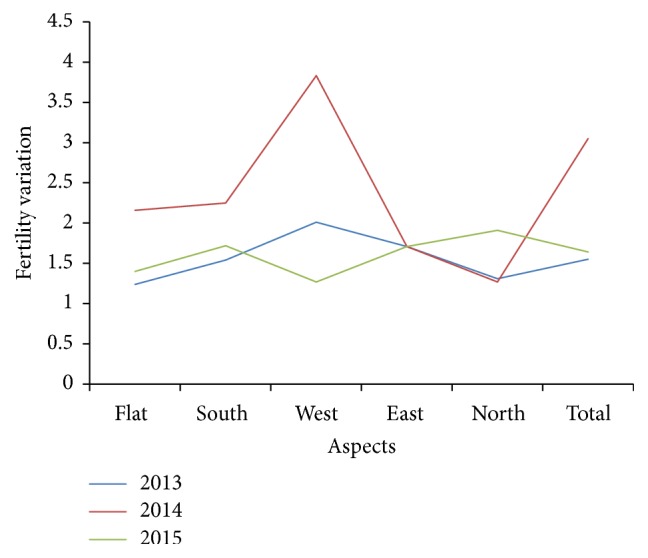
Fertility variations for aspectual populations and years.

Fertility variations changed for the aspect and years (Table 3 and Figure 2). Fertility variation was 1.55 for 2013, 3.05 for 2014, 1.64 for 2015, and 1.78 for polled years, while it was 1.24, 2.16, 1.40, and 1.42 for flat and 2.01, 3.83, 1.27, and 1.47 for west aspect (Table 3 and Figure 2). Data may be much affected by west population in 2014. These results showed that seed collecting years and/or stands could be important factors to transmit gene diversity from current to next generations as also emphasized [2] based on sustainable forestry practices. It was suggested that the sibling coefficient (Ψ) of natural stands as a heuristic rule of thumb could be set to three (Ψ = 3) and that of seed orchards could be set to two (Ψ = 2) [25]. Estimated fertility variations in the present study could be acceptable level for typical natural populations except of west population of 2013 (3.83) and for 2014 (3.05) (Table 3). These results emphasized importance of aspect and seed collection year.

The production of cones, flowers, pollen, fruits, and seeds has been used to estimate fertility and fertility variation in many plant species [18, 26, 27]. However, data collection on cone production was easier, cheaper, and more accurate than that of strobilus count. The tree keeps the cone in longer period than strobili in a year. So, data collection period is longer in cone than in strobilus counts as also emphasized [28]. In the present study, cone data was collected from only one population for each aspect. Therefore, it is needed to collect more data from large population on fertility variation to draw accurate conclusion.

The effective number of parents (*N*_*p*_) was mirrored to the fertility variation (Table 3). The effective number of parents was 84.3 (56.2% of census number) for combined year populations, while it was between 16.3 (54.2%, south) and 21.5 (71.8%, north). The results showed that the populations which had low effective number of parents (north and south) could be balanced to the ideal populations by traditional or genetic forest tending (e.g., removing of unproductive tree). Silvicultural practices such as natural regeneration are applied in good flowering year to obtain higher gene diversity for next generation. As presented in Table 3, gene diversity was similar (0.99) for the years in polled stands. However, it was ranged from 0.93 to 0.98 for years and stands (Table 3). Besides, while 2015 seemed to be good cone production year than 2013 (Table 1), fertility variation was similar (1.64 and 1.55) for the years (Table 3). The result implied that the gene diversity in good flowering year could be similar to the gene diversity in moderate or poor flowering years, which may be important for genetic researchers and natural regenerations of the species as also emphasized [2]. In contrast to years, stands had large fertility variation within year. For instance, it was between 1.24 (flat) and 2.01 (west) for 2013 (Table 3). It emphasized importance of aspect in selection of gene conservation areas and seed sources. North aspect could be taken into consideration in establishment and selection of seed sources and gene conservation areas based on fertility variation and gene diversity.

## 4. Conclusions

Aspect is one of the most important environmental factors for forestry practices because of its easy application for managers. It is used in many purposes in forestry from selection of seed sources and nursery practice to natural regeneration or forest establishment. Results of the study also emphasized importance of aspect in forestry practices such as preparing of silvicultural planning aspect level and management of gene conservation areas in watershed.

## Figures and Tables

**Table 1:** Averages $\left(\overset{-}{x}\right)$ and coefficient of variation (CV%) for number of cones for the aspects and years.

Aspects	Year						Total	
	2013		2014		2015			
	$\overset{-}{x}$	CV%	$\overset{-}{x}$	CV%	$\overset{-}{x}$	CV%	$\overset{-}{x}$	CV%
Flat	23.5	54.0	74.1	120.2	95.7	64.1	64.5	66.0
South	16.3	78.5	92.1	113.8	93.6	86.1	67.4	93.5
West	20.3	105.0	33.0	175.2	85.0	52.7	46.2	69.3
East	16.8	92.9	18.2	117.6	65.7	89.2	33.5	76.4
North	20.2	60.4	18.8	66.5	39.4	97.0	26.1	63.6
*Polled*	*19.4*	*79.4*	*47.2*	*155.3*	*75.9*	*81.0*	*47.5*	*88.6*

**Table 2:** Correlation coefficients for cone production among years.

*r*_*p*_^*∗*^	2014	2015	Total
2013	0.570	0.408	0.867
2014	—	0.292	0.894
2015		—	0.489

^*∗*^The correlations are significant at the 0.01 level.

**Table 3:** Fertility variation (Ψ), effective number of parents (*N*_*p*_), relative effective number of parents (*N*_*r*_), and gene diversity (GD) for the aspects and years.

Aspects	Year												Total			
	2013				2014				2015							
	Ψ	*N*_*p*_	*N*_*r*_	GD	Ψ	*N*_*p*_	*N*_*r*_	GD	Ψ	*N*_*p*_	*N*_*r*_	GD	Ψ	*N*_*p*_	*N*_*r*_	GD
Flat	1.24	23.4	80.7	0.98	2.16	12.5	46.4	0.96	1.40	21.5	71.6	0.98	1.42	21.1	70.3	0.98
South	1.54	18.7	64.8	0.97	2.25	13.3	44.4	0.96	1.72	17.5	58.3	0.97	1.84	16.3	54.2	0.97
West	2.01	14.5	49.9	0.97	3.83	7.57	26.1	0.93	1.27	23.7	78.9	0.98	1.47	20.5	68.2	0.98
East	1.71	16.3	58.4	0.97	1.71	12.9	58.4	0.96	1.71	17.0	58.5	0.97	1.57	19.1	63.8	0.97
North	1.31	22.2	76.7	0.98	1.27	21.2	78.5	0.98	1.91	15.7	52.5	0.97	1.39	21.5	71.8	0.98
*Polled*	*1.55*	*92.5*	*64.7*	*0.99*	*3.05*	*44.2*	*32.8*	*0.99*	*1.64*	*90.8*	*60.9*	*0.99*	*1.78*	*84.3*	*56.2*	*0.99*
